# Alcohol consumption and accentuated personality traits among young adults in Romania: a cross-sectional study

**DOI:** 10.1186/s13011-016-0080-3

**Published:** 2016-10-27

**Authors:** Cornelia Rada, Alexandru Teodor Ispas

**Affiliations:** 1 “Francisc I. Rainer” Anthropology Institute of the Romanian Academy, 8 Eroii Sanitari Avenue, O.P. 35, C.P. 13, Sector 5, 050474 Bucharest, Romania; 2 “Carol Davila” University of Medicine and Pharmacy Bucharest, 37 Dionisie Lupu Street, Bucharest, Romania

**Keywords:** Alcohol consumption, Young adults, Accentuated personalities, Demonstrativeness, Hyperthymia, Cyclothymia, Romania

## Abstract

**Background:**

Alcohol consumption (AC) has negative social and economic consequences, affects health, and can create dependence. As dependence is particularly difficult to cure, prevention is important. This study aimed to identify the frequency, quantity, occasions, reasons, type of AC, and correlation with accentuated personality traits among young adults in Romania.

**Methods:**

Participants were 1359 young adults aged 18–30 years (average age, 22.67 years; standard deviation [SD], 3.02 years) from urban environments including the main university centers. Several questionnaires covering issues such as health risk behavior (smoking, alcohol abuse, unprotected sex, sedentary lifestyles, unhealthy eating), aggression, personality, adaptability, cohesion, and communication were administered to participants between 2013 and 2014. Pearson’s chi-square tests and z-tests were used for the analyses.

**Results:**

Common reasons young adults first tried AC were curiosity (67.8 %), to be like peers (17.9 %), and adult influence (6.5 %). In terms of AC frequency, 72.5 % consumed alcohol only on special occasions/holidays, 19.4 % on weekends, 4.8 % three to four times per week, and 0.4 % on a daily basis. To overcome sexual/emotional inhibitions or for courage, 2.1 % of participants drank frequently and 23.5 % drank from time to time. AC most often occurred with a group of friends (62.3 %). For 9.7 % of participants, AC was a reason for poor concentration, or problems at work/school. At the time of interview, participants had consumed an average of 319.48 ml beer (SD, 1223.02 ml), 82.75 ml wine (SD, 385.39 ml) and 25.62 ml spirits (SD, 131.34 ml) in the previous week. AC was significantly higher in males (*p* < 0.01), and in participants aged 23–30 years (*p* < 0.05). AC was influenced by six accentuated personality traits: Demonstrativeness, Hyper-perseverance, Uncontrollability, Hyperthymia, Cyclothymia, and Exaltation (*p* < 0.01).

**Conclusions:**

AC was relatively high, especially among young men, peer groups, and young adults who had problems socializing. AC also correlated with some accentuated personality traits. Therefore, public health education programs should be targeted for these categories.

## Background

Alcohol consumption (AC) has negative social and economic consequences, affects health, and can create dependence [1, 2]. Dependence is particularly difficult to cure, and prevention is important.

The 2014 global status report on alcohol and health indicated that worldwide, 3.3 million deaths result from harmful use of alcohol every year, representing 5.9 % of all deaths [3]. In Romania, as in other countries, alcohol is the most prevalent substance used among youth in early and late adolescence [4].

The average adult (15+ years) AC per capita in Romania in 2003–2005 was 12.8 l of pure alcohol (including unrecorded consumption), and in 2003–2005 was 14.4 l. This represents higher consumption than in the World Health Organization (WHO) European Region.

In 2010, the 12-month prevalence estimates of alcohol use disorders (in people 15+ years) in Romania were 3.8 % in males and 1.1 % in females. This was more than three times lower than in the WHO European Region. The prevalence of heavy episodic drinking (at least 60 g or more of pure alcohol on at least one occasion in the past) for people aged 15+ years was 13.8 % for males and 1.7 % for females, with age-standardized death rates (per 100,000 population aged 15+ years) being 65 % liver cirrhosis and 18.9 % road traffic accidents [5].

The European School Survey Project on Alcohol and Other Drugs (ESPAD) underpins the development of policies, strategies and interventions for high school students. In 2011, Romania had the fourth strongest participation in the project, which involved institutions such as the National School of Public Health, Management, and Professional Development, the National Anti-Doping Agency (NAA), the Ministry of Education, Research, Youth, and Sports, and the county school inspectorates. These agencies cooperated with school principals and head teachers of selected classes.

In 2011, 2770 Romanian high school students aged 16 years participated in the ESPAD. However, 9 % of parents and 2 % of students refused to participate, which were the highest percentages of refusal among all ESPAD countries (European average of 1 % refusal). Participating Romanian adolescents had an abstinence level of 9.2 %, although 33 % had experienced drunkenness, a rate lower than in 2003 and 2007, and 14 % lower than the average of the ESPAD countries. At least once in their lifetime, 3.2 % of participating adolescents had consumed alcohol with pills [6].

In 2013, the Romanian NAA conducted a General Population Survey covering tobacco, alcohol and drug consumption. That survey used standard methodologies which allowed data comparisons at a European level. Survey participants were 5700 people aged 15–64 years (selected nationally), and 1500 young people aged 15–34 years selected from Bucharest, Romania’s capital city. That study found 6.2 % of participants reported daily consumption, with the age of onset below 18 years. The main reasons for AC were relaxation, joy, energy, and entertainment (around 25 %) followed by group membership (“Because my group of friends drink,” 11.5 %) [7].

In Romania, AC tends to be higher in regions with grapes and fruit trees. For example, compared with Romanian youth overall, youth in Piteşti drink more. In addition, parents generally have a permissive attitude towards youth drinking, and sometimes even facilitate early initiation of alcohol. Therefore, educational projects focused on healthy AC norms have involved parents [8].

It is difficult to estimate the actual AC in Romania, as large amounts of spirits and wine are produced and consumed in households [8]. In the hill regions, where there are many orchards, most inhabitants (rural and urban) have the necessary equipment at home to prepare spirits such as ţuică, rachiu, and palincă. These beverages are brandy made from plums or a mixture of fruits, with the alcohol content by volume ranging from 30 to 40 % to over 50 %. Wine is prepared in households in the viticultural areas. Besides industrial bottling and marketing, these beverages are used for family consumption and trade in small agricultural markets.

AC prevention and limitation are important in reducing hazardous drinking (occasional binge drinking) with an increased risk for harm, harmful drinking (pattern of drinking) that has a higher risk, and dependent drinking (the most serious problem) [9]. Ideally, AC should be initiated as late as possible, at least 5–6 years after coming of age (18 years), to reduce the risks for developing related health problems and addiction [10]. Some studies have shown that a proportion of those seeking treatment for alcohol abuse began drinking in adolescence [11]. A study conducted in the USA in 2001–2002 with a sample almost 35,000 adults aged 18 years and older found that the younger the age at which people started to drink, the greater their likelihood of developing alcohol dependence within 10 years of drinking onset and before age 25 years [12].

Personality can also make a valuable contribution to our understanding of behavioral problems such as AC, and addiction in a broader sense. In 1957, Syme stated that “Careful assessment of the literature since 1936 indicates that there is no warrant for concluding that persons of one type are more likely to become alcoholics than persons of another type” [13]. Miller (1976) asserted that personality traits observed in alcoholics were the result and not the cause of their alcoholism [14]. McClelland and colleagues [15] found that heavy drinkers in college were characterized by a high need for power. MacAndrew [16] conducted a study with young people aged 16–21 years who were treated for alcohol problems, and concluded that personality type preceded alcoholism, rather than resulting from it. Hoffman et al. [17] and Loper et al. [18] identified that before and after clinical diagnoses of alcoholism, participants were characterized by anti-social impulsiveness. In AC, personality may be a risk factor, but should not be viewed as a cause and effect relationship (e.g., if you have “X” or “Y” personality you will have problems with alcohol). Jessor and Jessor found that people who were oriented to prosocial activities were unlikely to abuse drugs or alcohol whatever their personality type [19].

Peele synthesized personality traits that may lead to alcoholism, including excessive behaviors such as antisocial outlook (including a lack of achievement orientation), lack of ability to create intimacy, tendency to seek feelings of power through drinking, use of alcohol to palliate depression or anxiety, and tendency to feel one does not have control over one’s life and define oneself externally. Other factors are also important, including family background, age, sex, ethnicity, cultural background, or circumstantial factors that may cause these personality characteristics to manifest in excess and cause individual dysfunctional [20].

Alcohol-dependent inpatients treated under standardized conditions and who relapsed during treatment had significantly higher scores on “obsessions” and “drinking control and consequences” compared with patients who were abstinent [21]. A study conducted in 2013–2014 with 267 medical students in Cluj Napoca, Romania, found that personality traits such as extroversion and openness to new experiences were associated with drug consumption and AC [22]. Other studies have shown connections between alcohol consumption, personality, and sociocultural factors [23–25].

### Objectives

In the above context, the present study aimed to survey Romanian young adults to identify:

- the frequency, quantity, occasions, reasons, and type of AC,
- if AC was an impediment to work activities or study,
- the correlation between AC and accentuated personality traits,
- the influence of sex and age on AC,
- family AC and the influence on young adults,
- psychometric qualities of the Schmieschek Questionnaire for accentuated personality traits in the current cultural and temporal context.

This study is important as there are few English-language studies from a multidisciplinary perspective focused on AC in Romania. Our study will fill a gap in the specialized literature. This is also the first study using the Schmieschek Questionnaire to determinate the correlation between AC and accentuated personality traits. Understanding accentuated personality traits is important as they are possible risk factors for AC, and may be helpful in clinical practice to identify patients at risk for relapse, as well as supporting psychotherapy. This study may be a first step in revalidating the Schmieschek questionnaire in the present context. Accentuated personality traits were considered insufficient and early diagnosis may help to prevent the appearance of clear symptoms.

Our data on AC and variables such as sex, age and personality traits may help to inform public health policies on prevention and support, and in designing evidence-based prevention programs rather than general prevention programs.

## Methods

### Design and sampling

We conducted a quantitative cross-sectional study between 2013 and 2014 with 1359 young adults aged 18–30 years (average age, 22.67 years; standard deviation [SD], 3.02 years). Participants were randomly selected from urban areas in Romania including the main university centers: Timișoara (Timiş county), Zalău (Sălaj county), Baia Mare (Maramureş county), Cluj-Napoca (Cluj county), Târgu Mureş (Mureş county), Sibiu (Sibiu county), Braşov (Braşov county), Piteşti (Argeş county), Craiova (Dolj county), Iaşi (Iaşi county), Tulcea (Tulcea county), Constanţa (Constanţa county), and Bucharest (Romania’s capital city).

Participants (74.6 %) who attended an educational institution (pre-university, university, post-university) completed the questionnaires during seminars. Other participants were recruited from among employees of educational or cultural institutions or businesses, and completed the questionnaires at home. Table 1 presents the structure of the surveyed sample.

**Table 1:** Participants’ sociodemographic and family characteristics (*N* = 1359)

Sociodemographic data	Number	Percent
Sex		
Male	533	39.2
Female	826	60.8
Age groups (years)		
18–22	724	54.5
24–30	604	45.5
Marital status		
Unmarried (single)	1100	80.9
Married	174	12.8
Consensual union over 1 year	85	6.3
Education		
Elementary/high school	29	2.1
Lyceum/some years in University	1009	74.3
University degree	321	23.6
Housing		
Lives with the family of origin (family into which was born and grew up)	928	68.3
Lives alone	64	4.7
Lives with the family created through and following marriage or consensual union	367	27.0

None of the participants or their family members had diagnoses of psychiatric illnesses or psychological problems.

Each locality had two or three people responsible for data organization, collection, and verification. Verification of completed questionnaires was performed with participants face-to-face. Each person responsible for checking the questionnaires had expertise in sociology, psychology, or medicine. The response rate was 100 %.

### Measurements and questionnaire design

A 60-item omnibus-type questionnaire was used to collect sociodemographic data, relevant information about participants’ families, and items related to health risks (smoking, alcohol abuse, unprotected sex, sedentary lifestyle, unhealthy eating, and violence). Participants were also weighed and their height was measured.

Participants completed three questionnaires: the Aggression Questionnaire [26], the Schmieschek Questionnaire for accentuated personality traits (88 items) [27] and 13 items from the Jenkins Activity Survey [28]. In addition, participants completed the Family Adaptability and Cohesion Scale (FACES) IV Package, containing eight scales; six from FACES IV (42 items), and the Family Communication and Family Satisfaction scales, each with 10 items (acquired by the author under license) [29].

### Data management and statistical analysis

The Schmieschek Questionnaire emphasizes aspects leading to the diagnosis of accentuated personality traits: Demonstrativeness, Hyper-exactness, Hyper-perseverance, Uncontrollability, Hyperthymia, Dysthymia, Cyclothymia, Exaltation, Anxiety, and Emotivity. The questionnaire was translated and adapted in Romania in 1975 by Nestor. Accentuated personality traits are between normality and pathology and have a series of special features above the general population average; these personality structures can adapt to favorable environments but can also decompensate under demanding conditions. In the questionnaire, personality traits may be considered accentuated when the number of significant or symptomatic responses in a group (pre-established by the author) exceeded 50 % [30–32].

The maximum score that can be obtained for each scale is 24, with a value of 12 representing the cut-off score for an accentuated behavior. The scores used to delineate the categories in our study were: 0–11 = normal, 12–17 = accentuated, 18–23 = strongly accentuated, and 24 = significantly accentuated. The internal consistency for the overall Schmieschek questionnaire (88 binary items) was good, with a Cronbach’s alpha value of 0.82. None of the items used would substantially affect reliability if they were deleted. However, the internal consistency for each subscale was low, with Cronbach’s alpha values ranging from 0.212 to 0.651 (Demonstrativeness, 0.367; Hyper-exactness, 0.651; Hyper-perseverance, 0.286; Uncontrollability, 0.505; Hyperthymia, 0.212; Dysthymia, 0.212; Cyclothymia, 0.478; Exaltation, 0.314; Anxiety, 0.572; and Emotivity, 0.415).

The present study focused on the results of the analysis of the Schmieschek Questionnaire and seven AC items:

1. For what reason did you first consume alcoholic drinks? Response options: a) To be like my friends/classmates/colleagues; b) I was bored; c) Curiosity; d) Adult influence.
2. How often do you have an alcoholic beverage? Response options: a) Daily; b) three or four times a week; c) On weekends; d) Only on special occasions/holidays.
3. Do you ever drink to overcome sexual or emotional inhibitions or for courage? Response options: a) Almost never; b) Sometimes; c) Often.
4. Has alcohol ever been a reason for poor concentration or trouble at work/school? Response options: a) No; b) Yes.
5. How many ml of the following alcoholic drinks have you consumed in the last 7 days? If you have not had any, answer “0.” Response options: a) Beer; b) Wine; c) Spirits/liquor (e.g., vodka and so on).
6. You most often consume alcoholic beverages: a) With family; b) With a group of friends; c) With my best friend; d) With my boyfriend/girlfriend; e) Alone.
7. In your family, alcoholic beverages are consumed: a) Daily; b) Weekly; c) On different occasions; d) Almost never or never.

We used Pearson’s chi-square tests for the analyses. All analyses were performed with SPSS Version 15. Demographic variables used in the statistical analyses were sex and age group. Differences or associations were considered statistically significant at *p*-values between <0.0001 and <0.05. After chi-square tests, we used column proportions z-tests to determine the relative order of the column categorical variables in terms of the proportions of the row categorical variables, to determine which rows and columns were responsible for the relationship.

## Results

**Fig. 1:**
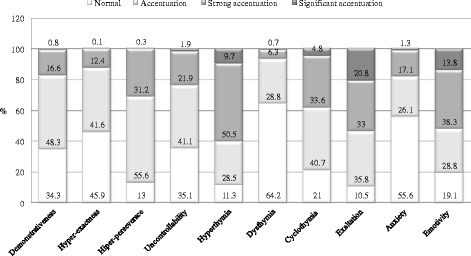
Distribution of participants by accentuated personality traits

For the Schmieschek Questionnaire, Exaltation, Hyperthymia, and Hyper-perseverance showed the lowest percentages of normality. Dysthymia, Anxiety, and Hyper-exactness showed the highest percentages of normality. Exaltation, Emotivity, and Hyperthymia were significantly accentuated (Fig. 1).

**Fig. 2:**
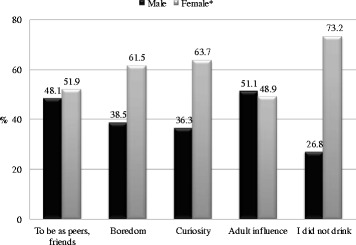
Reasons why young people first tried alcohol, by sex

Participants first tried alcohol because of curiosity (67.8 %), to be like peers (17.9 %), adult influence (6.5 %), and boredom (4.8 %). Overall, 3 % of participants never drank alcohol. The percentage of women who had never consumed alcoholic drinks was much higher than that of men. The most common reason women first tried alcohol was curiosity followed by boredom, whereas the most common reasons for men were adult influence and to be like peers/classmates/colleagues (chi square = 16.47, df = 3, *p* < 0.01) (Fig. 2). The comparisons of column proportions indicated that a greater proportion of males used AC to be like peers/friends than because of curiosity. In contrast, more females used AC for curiosity than to be like peers/friends.

The main reason for first trying alcohol in participants with strongly accentuated Cyclothymia was boredom, and in those with accentuated Cyclothymia was curiosity (chi square = 22.921, df = 9, *p* < 0.01). Participants with strongly accentuated Hyperthymia first tried alcohol because of curiosity and adult influence (chi square = 30.467, df = 9, *p* < 0.001) (Table 2).

**Table 2:** Reasons why young people first tried alcohol consumption by cyclothymia and hyperthymia

Personality traits		The reasons for which young people tried first AC			
		To be as peers	Boredom	Curiosity	Adult influence
Cyclothymia^a^	Normal	28.0	21.5	17.8	29.5
	Accentuation	40.7	32.3	41.9	34.1
	Strong accentuation	26.4	43.1	35.4	33.0
	Significant accentuation	4.9	3.1	5.0	3.4
Hyperthymia^b^	Normal	16.5	18.5	9.5	9.1
	Accentuation	32.1	29.2	26.9	36.4
	Strong accentuation	40.7	35.4	54.4	46.6
	Significant accentuation	10.7	16.9	9.1	8.0

^a^Pearson’s chi-square test = 22.921, *p* < 0.01, ^b^Pearson’s chi-square test = 30.467, *p* < 0.001; all df = 9

A comparison of column proportions indicated that a significantly larger proportion of participants who scored normal for Cyclothymia first tried AC to be like peers or due to adult influence than who first tried AC for curiosity. For those classified as strongly accentuated Cyclothymia, the proportion who first tried AC for curiosity was significantly larger than that who first tried AC to be like peers. A significantly higher proportion of participants classified as strongly accentuated Hyperthymia first tried AC for curiosity than to be like peers or due to boredom. In participants with normal Hyperthymia, more participants first tried AC to be like peers than due to curiosity.

**Fig. 3:**
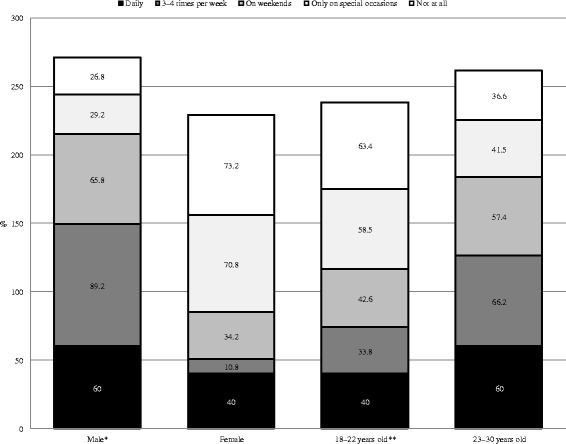
Frequency of alcohol consumption by sex and age group

In terms of frequency of AC, 72.5 % of participants only used alcohol on special occasions or holidays, 19.4 % on weekends, 4.8 % three/four times per week, and 0.4 % consumed alcohol daily. The frequency of AC was higher for men (chi square = 190.74, df = 4, *p* < 0.001) and for the group aged 23–30 years (chi square = 34.21, df = 4, *p* < 0.001) compared with women and those aged 18–22 years. The comparisons of column proportions indicated that a greater proportion of males used AC three/four times per week than on weekends, special occasions/holidays, or did not use AC. More females used AC only on special occasions than on weekends or three/four times per week. More participants in the group aged 18–22 years used AC on special occasions than three/four times per week or on weekends. In the group aged 23–30 years, the proportion that used AC three/four times per week was significantly greater than those who did not use AC or used AC on weekends (Fig. 3).

Daily AC was common in participants with accentuated Uncontrollability (chi square = 39.85, df = 9, *p* < 0.001) and with strongly accentuated Exaltation (chi square = 26.58, df = 9, *p* < 0.01). The column proportions z-test indicated that a significantly greater proportion of participants with normal Uncontrollability only used AC on special occasions than that used AC on weekends. More participants with strongly accentuated Uncontrollability used AC on weekends than on special occasions or three/four times per week. The proportion of those with normal Exaltation who reported AC 3–4 times per week was greater than those who reported AC only on special occasions or weekends (Table 3).

**Table 3:** Alcohol consumption frequency by uncontrollability and exaltation

Personality traits		AC frequency			
		Daily	3–4 times per week	On weekends	Only on special occasions
Uncontrollability^a^	Normal	20.0	33.8	25.9	37.3
	Accentuation	60.0	46.2	36.1	41.7
	Strong accentuation	20.0	15.4	35.4	19.6
	Significant accentuation	0	4.6	2.7	1.4
Exaltation^b^	Normal	0	24.6	12.5	9.3
	Accentuation	40.0	36.9	39.2	34.7
	Strong accentuation	60.0	18.5	32.3	33.6
	Significant accentuation	0	20.0	16.0	22.3

^a^Pearson’s chi-square test = 39.85, *p* < 0.001, ^b^Pearson’s chi-square test = 26.58, *p* < 0.01; all df = 9

**Fig. 4:**
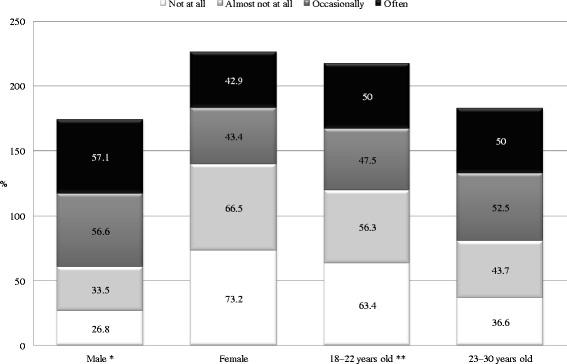
Alcohol consumption to overcome sexual or emotional inhibitions or for courage, by sex and age group

To overcome sexual or emotional inhibitions or for courage, 2.1 % of participants drank frequently, and 23.5 % drank from time to time. Using AC to overcome sexual or emotional inhibitions or for courage was more common in males (chi square = 60.08, *p* < 0.001, df = 3) and in the group aged 23–30 years (chi square = 9.09, *p* < 0.05, df = 3). The z-test indicated that a greater proportion of males occasionally used AC to overcome sexual or emotional inhibitions than who did not use AC for this purpose. In contrast, more females almost never or never used AC to overcome sexual or emotional inhibitions than who occasionally used AC for this purpose. In addition, a greater proportion of the group aged 18–22 years almost never used AC to overcome sexual or emotional inhibitions than that occasionally used AC. Significantly more participants in the group aged 23–30 years occasionally used AC to overcome sexual or emotional inhibitions than who almost never used AC for this purpose (Fig. 4).

By personality trait classification, the highest percentages of participants who occasionally used AC to overcome sexual or emotional inhibitions or for courage were those with accentuated Demonstrativeness (chi-square = 9.76, df = 3, *p* < 0.05), Hyper-perseverance (chi-square = 8.49, df = 3, *p* < 0.05), and Uncontrollability (chi-square = 19.97, df = 3, *p* < 0.001). A greater proportion of those with accentuated Demonstrativeness and significantly accentuated Uncontrollability occasionally or often used AC to overcome sexual or emotional inhibitions than who almost never used AC for that purpose. In addition, significantly more participants with strongly accentuated Hyper-perseverance or normal Uncontrollability almost never used AC to overcome sexual or emotional inhibitions than who used AC occasionally or often for that purpose (Table 4).

**Table 4:** Alcohol consumption to overcome sexual or emotional inhibitions or for courage, by Demonstrativeness, Hyper-perseverance, and Uncontrollability

Personality traits		AC in order to escape from sexual or emotional inhibitions, to take courage	
		Almost not at all	Occasionally, often
Demonstrativeness^a^	Normal	34.7	33.0
	Accentuation	46.3	53.4
	Strong accentuation	17.8	13.5
	Significant accentuation	1.1	0
Hyper-perseverance^b^	Normal	12.1	15.2
	Accentuation	54.2	58.6
	Strong accentuation	33.3	26.1
	Significant accentuation	0.4	0
Uncontrollability^c^	Normal	37.1	28.2
	Accentuation	40.0	43.4
	Strong accentuation	21.9	24.4
	Significant accentuation	1.0	4.0

^a^Pearson’s chi-square test = 9.76, *p* < 0.001, ^b^Pearson’s chi-square test = 8.49, *p* < 0.01, ^c^Pearson’s chi-square test = 19.97, *p* < 0.001; all df = 3

**Fig. 5:**
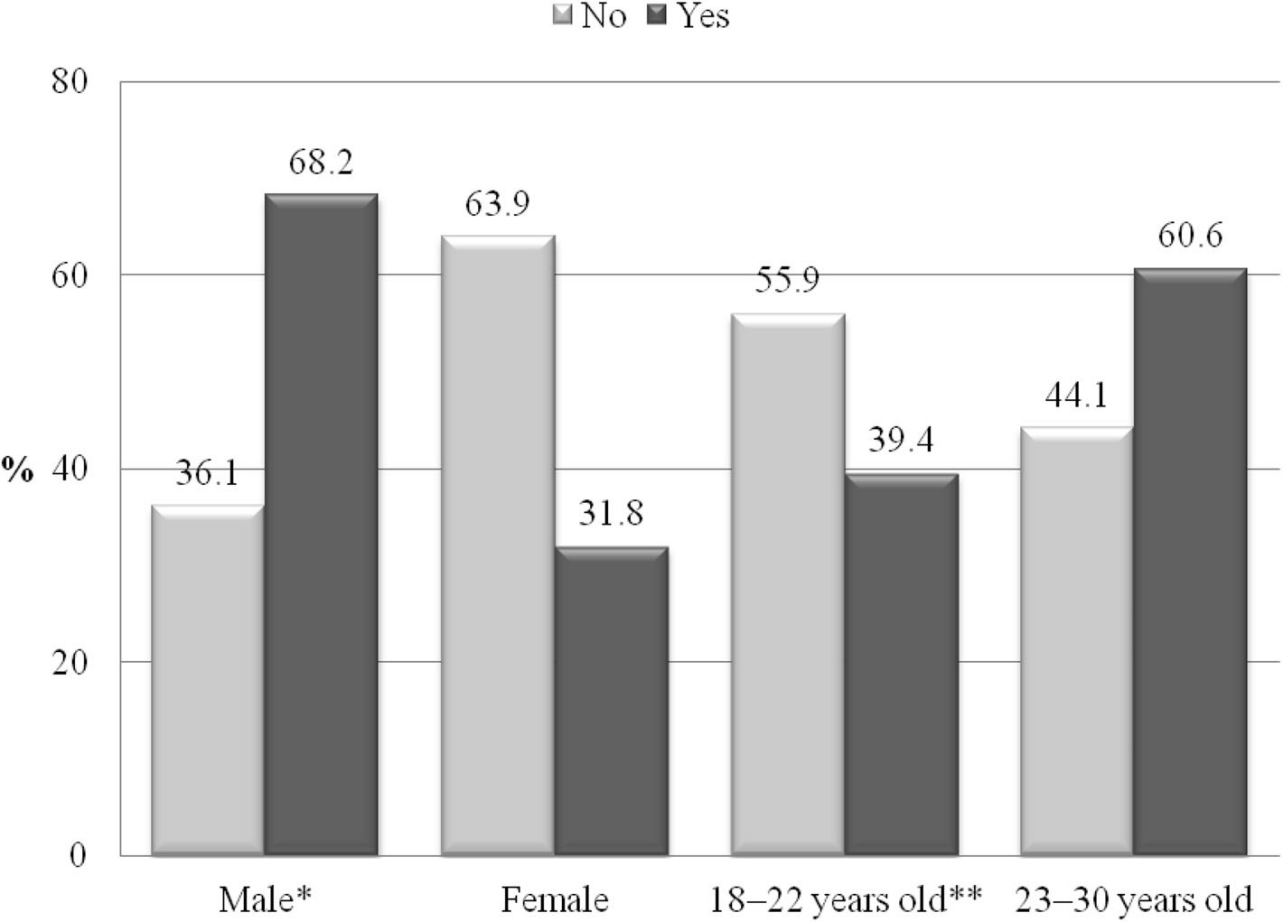
Alcohol consumption as the reason for poor concentration or problems at school/work, by sex and age group

For 9.7 % of participants, AC was a reason for poor concentration, and problems at work or at school, with this being more common in males (chi square = 51.44, df = 1, *p* < 0.001) and those aged 23–30 years (chi-square = 13.09, df = 1, *p* < 0.001) (Fig. 5).

The comparisons of column proportions indicated that for males and the group aged 23–30 years, more participants reported AC as a reason for poor concentration or problems at school/work than who said AC was not the cause of poor concentration or problems at school/work. In contrast, for females and the group aged 18–22 years, the use of AC was not the cause of poor concentration or problems at school/work in the majority of participants.

A noteworthy correlation was between significantly accentuated Demonstrativeness and poor concentration or problems at work/school, with 90.9 % of these participants reporting such problems after AC (chi-square = 18.95, *p* < 0.001, df = 3). No clear associations were found between AC and these problems for the other accentuated personality traits.

At the time they were interviewed, participants reported that in the last week, they consumed an average of 319.48 ml of beer (SD, 1223.02 ml), 82.75 ml of wine (SD, 385.39 ml), and 25.62 ml of spirits (SD, 131.34 ml). Overall, 26.41 % of participants consumed beer, 15.4 % wine, and 10.82 % consumed spirits. All three categories of drinks were consumed by 3.16 % of participants. Males had higher AC, with over 6.2 times the amount of beer, 3.5 times the amount of wine, and 3.8 times the amount of spirits than females.

Most often, AC occurred with group of friends (62.3 %), followed by with family (19.1 %), and then with a partner or best friend (10.7 %). Males consumed alcoholic beverages most often alone, followed by with a group of friends; whereas females consumed alcohol most often with family, followed by with their boyfriend (chi-square = 85.34, df = 5, *p* < 0.001). The z-test indicated that a greater proportion of males used AC alone than with family, with a group of friends, or who did not use AC. More females used AC with family or with their boyfriend than alone or with their best friend. The proportion of participants who did not use AC was greater than those who used AC alone or with family.

Participants aged 18–22 years consumed alcoholic beverages most often with their best friend, followed by a group of friends; whereas those aged 23–30 years consumed alcohol most often with their boyfriend/girlfriend, followed by with family (chi-square = 15.008, df = 5, *p* < 0.05) (Table 5). In the group aged 18–22 years, a greater proportion of participants did not use AC than who used AC with family or with a boyfriend/girlfriend. Significantly greater proportions of participants in the group aged 23–30 years used AC with family or with a boyfriend/girlfriend than the proportion who did not use AC.

**Table 5:** Where alcohol consumption occurred most often, by sex and age group

Sex, age groups	In the family	With the group of friends	With the best friend	With the boyfriend/girlfriend	Alone	Not at all
Male^a^	23.1	47.5	40.7	18.4	57.7	19.8
Female	76.9	52.5	59.3	81.6	42.3	80.2
18–22 years old^b^	47.7	55.9	59.3	43.7	53.8	66.7
23–30 years old	52.3	44.1	40.7	56.3	46.2	33.3

^a^Pearson’s chi-square test = 85.34, *p* < 0.001, ^b^Pearson’s chi-square test = 15.008, *p* < 0.05; all df = 5

Participants reported AC with family on special occasions (60.2 %), weekly (13.2 %), daily (4 %), and not at all (22.6 %). Compared with males, twice as many females stated that in their family, alcohol was almost never or never consumed; at the same time, AC on special occasions was over 1.5 times higher in females than males. We found a correlation between AC with family and AC frequency (Chi-square = 185.63, df = 12, *p* < 0.001). Over 75 % of participants who reported almost never or never using AC and over 60 % of those who only used AC on special occasions/holidays belonged to families with the same AC frequency.

## Discussion

Only a small percentage (3 %) of participants reported that they did not consume alcoholic beverages. Our findings are consistent with those published by Bucur et al. [33], who studied 933 young people (aged 14–30 years, average age 22.12 ± 2.78 years) living in Mureş county and in neighboring Romanian counties, and found 3.75 % abstinence from alcohol. In our study, abstinence was more than four times lower compared with data collected by Shah et al. [34] from 2710 medical students in the USA.

Over 70 % of the young people in our sample only consumed alcohol on special occasions or holidays, indicating that AC was relatively low in comparison with that of young people reported in some studies in England [35–37]. For all categories of alcoholic beverages, the amount consumed was much higher in males, and suggested that females preferred wine over beer or spirits. This factor should be investigated in more detail in future to determine the kinds of beverages associated with different types of drinkers: severe, moderate, and occasional drinkers (AC for energy). Other studies found that wine consumption was not strongly associated with smoking habits and problems caused by AC [38]. Further studies should be conducted to clarify which variables have a greater impact on these associations.

Previous studies have shown that the prevalence of AC and of problems caused by drinking were higher among men than women [39–41]. Our study also found males drank more frequently than females, drank more often to overcome sexual or emotional inhibitions or for courage, and had over twice as many AC-related problems in concentration at work/school. In addition, the percentage of females who did not consume alcohol at all was 2.7 times higher than that of males. Interventions to raise awareness among parents, schools, and society in general are necessary, particularly as currently, there are more permissive messages about AC for men whereas women receive higher social sanctions for drinking.

Studies have shown that people who have ordered and well-structured daily lives (e.g., who are in a stable couple, are parents, and have a paid job) tend to drink less than people who are not involved in a relationship, have no children, and no job [42]. Our sample included 12.9 % of participants who were married, 8.3 % who had been in a consensual union for more than 1 year, 4.6 % who lived alone, 8.9 % who had children, and 2.6 % who were unemployed and not attending a higher education institution. We did not identify significant correlations between AC and these variables. However, we found that young people aged 23–30 years, most of whom did not live with parents or a partner, drank more often, drank more frequently to overcome sexual/emotional inhibitions of for courage, and had greater AC-related problems concentrating at work or school. This indicates a positive impact of family life, which imposes a certain structure and order. It is also important that future studies consider the advantages and disadvantages for young adults living on a university campus.

A survey conducted in Germany, Bulgaria, Poland, and the UK (2007) and in Slovakia (2008) with 2529 university student freshmen (mean age 20.37 years) found an association between perceived stress and depressive symptoms and problem drinking [43]. In the context of AC, the problem may not be stress, but rather the coping mechanism for stress [44].

In our sample, the types of accentuated personality traits influenced attitudes and behavioral patterns related to AC to a certain extent; for example, AC was correlated with Uncontrollability and Exaltation. In particular, significantly accentuated Exaltation was significantly correlated with AC. It might be that those whose emotional sphere oscillates between enthusiasm and despair or euphoria and discouragement use AC as a coping mechanism.

We did not identify a significant correlation between significantly accentuated Anxiety and AC as we expected, which was inconsistent with previous studies that found a connection between high anxiety sensitivity and AC used as a coping mechanism [45]. In our study, accentuated and strongly accentuated Demonstrativeness were much lower than the levels found in a previous study with adolescents [46]. This suggests that with age, the “hormone storm” decreases, acceptance of body metamorphoses increases, and the nature of relationships with the outside world are reshaped, leading to a decrease in the need to attract attention through demonstrative behaviors, particularly those on the ludic register (bravado, lies and so on).

Among participants who had AC-related problems with concentration, most belonged to the category with significantly accentuated Demonstrativeness. This group drank more frequently to overcome inhibitions. The desire to be noticed and accepted by others is a need experienced by all people; however, the manifestation of this need and the way in which attention is sought are important. For example, Demonstrativeness in art is necessary and an advantage, whereas maladaptive manifestations of Demonstrativeness are represented by AC, drugs, or membership of certain subcultures.

Participants with Hyper-perseverance and Uncontrollability drank more often to overcome inhibitions. People with uncontrollable personality traits tend to be pleasure-driven and have a low capacity to comply with an organized lifestyle or diet, which might explain why AC was more frequent in that group. The profile of Hyper-perseverance (abnormal perseverance with increased susceptibility, stubbornness, anxiety, and fear) is a good fit for AC.

Our findings showed that people in the Hyperthymia and Cyclothymia categories most commonly drank for the first time out of curiosity or boredom. Previous studies have reported that hyperthymic people are predisposed to alcohol misuse [47, 48]. It may be that characteristics of people with hyperthymic tendencies, such as cheerfulness, exuberance, intrusiveness, lack of inhibitions, overconfidence, grandiosity, and high energy levels mean they more often experience states of boredom and curiosity, which they attempt to resolve with AC. The affective lability of people with cyclothymic traits (oscillation between contradictory emotional states) means they may engage in AC due to boredom, particularly if they experience feelings of worthlessness, isolation, loneliness, and minor episodes of depression.

Generally, people drink for a reason. AC has direct chemical effects on the body, as well as indirect effects such as reducing psychological tension, improving mood, and acceptance by peers. In our sample, AC most commonly occurred with a group of friends, indicating AC was used as a way to socialize. AC with a group of friends was predominant in males, whereas in females, AC was more common with boyfriends and with family. This suggests that men tend to be social drinkers, and women tend to drink more in dyads or under family supervision. In Romanian society, in general, women are more heavily criticized than men if they are seen in public smoking or consuming alcoholic beverages.

The reasons given by young people in our sample for first consuming alcohol were similar to those revealed in other studies [49, 50]. Curiosity was a common reason for first trying alcohol. Females first tried alcohol more often out of curiosity or boredom and males mostly tried AC to be like their peers/friends. Therefore, it may be that women tend to drink to feel the effects of alcohol, have fun, improve their mood, relax, or to escape boredom, whereas men drink to be accepted by their peers.

In the youngest age group (18–22 years), AC was most common with a group of friends, whereas in the older age group (23–30 years), AC was most common with family. This is consistent with the results of previous studies, and suggests that age-related changes of status influence the preferred medium of AC [51]. For younger participants, AC was a modality to socialize with a group, and for older participants, was a modality of relaxation with a smaller group (i.e., family). Similar to other studies, we noted that pressure from peers/friends and curiosity may be predisposing factors for AC [52].

The AC pattern of young people is influenced by that of their families [53, 54]. AC on different occasions occurred with family in over half of the cases. Young people’s daily and weekly AC occurs with family in similar percentages. This highlights the importance of the family environment in terms of the formation of attitudinal and behavioral patterns in general, and of those concerning AC in particular [55, 56]. Parents should also monitor their children, as some young people tend to drink at home when parents are away [57].

The limitation of AC in young people is a major responsibility, particularly as studies have found correlations between AC and unprotected sex, which increases the risks of unplanned pregnancies or of sexually transmitted diseases [58].

As adolescents’ brains are still developing, alcoholic beverages should not be served or sold to people aged under 21 years, or even under 24 years. The age of 18 years represents the coming of age in Romania; however, as the limit that regulates the selling of cigarettes and alcoholic beverages, this age is too young. Furthermore, research in Piteşti (Argeş county) in Romania has shown that respect for the law is relatively low [59].

An initiative that contributed to the decline of AC in the UK was *Challenge 21* and *Challenge 25*, introduced by the British Beer and Pub Association (BBPA). Despite the fact that the minimum age to buy alcohol and cigarettes in the UK is 18 years, customers attempting to buy age-restricted products are asked to prove their age if, in the retailer’s opinion, they look under 19, 21, or 25 years [60]. This requires legislative measures as well as public health educational programs with clear explanations of the reasons why AC is not advisable, at least until that age. In addition, the explanations parents or teachers provide to young people usually include reasons such as “you are not allowed to drink because you are a kid,” “because it is illegal,” “when you earn your own money you could drink,” and so on. These statements do not offer helpful information about the harmful potential of alcohol, especially in this period of growth and development.

Another way to reduce AC through acknowledging negative consequences is to involve pharmacists. Pharmacists could disseminate brochures, flyers, or other information materials with attractive designs and friendly illustrations/text on this topic to their clients. In the UK, a randomized controlled trial was conducted with people who consumed alcohol that involved the pharmacist community, based on a brief intervention [61].

Teaching young people about healthy coping strategies may be helpful in reducing AC, including listening to music, going out with a friend (e.g., shopping, a movie, dining), spending time outdoors, playing with a pet, praying, or going to church.

### Limitations

Our study has some limitations. The population in our study cannot be taken as representative of all young adults in Romania. However, it can be considered representative of young people in higher education institutions, who live with parents, and who are childless. We did not collect information on participants’ regular daily schedules (e.g., how they spent their leisure time). In addition, our category “drink alcohol only on weekends” did not capture occasional binge drinking, drunkenness on weekends/at parties, or recreational alcohol users. Further studies are necessary to clarify the variables that have greatest impact on AC. Future studies should also use a qualitative design, such as individual or focus group interviews.

It is possible that our participants tended to conform to perceived expectations, and therefore AC was identified at lower levels than actual levels. In Romania, studies on alcohol and drugs with participants who are not known consumers (non-hospitalized, have not requested support) are less common compared with other countries such as the UK. In addition, the specific cultural matrix of the former communist bloc in Romania should be considered, as this imposed population conformism. Our participants were born around the time a democratic, liberal system was introduced (1989) and were educated by parents and teachers who were brought up in a society where socially desirable behavior was mandatory. The stigmatization of people who use alcohol excessively, especially women, might therefore have caused underreporting of AC.

In addition, data for the Schmieschek Questionnaire must be interpreted prudently due to the low internal consistency of the subscales. We did not collect information on the role of the family environment and parental control on AC of young adults.

A study conducted in Romania with a representative sample comprising 1062 parents and including qualitative data from 26 interviews with parents who came from families where at least one parent was an alcoholic, identified lower probability of AC in adolescents and adults who had emotionally and affectively available parents in the first 5 years of life, along with consistent and sustained discipline [62]. A study conducted in Germany with 59 inpatients aged 24–70 years who were addicted to alcohol found a high prevalence of insecure attachment [63].

## Conclusions

Public health policies, and educational methods and policies should consider the risk factors and vulnerability factors that influence the way young people engage in AC. Our study suggested that some accentuated personality traits (Demonstrativeness, Hyperthymia, Cyclothymia, Hyper-perseverance, and Uncontrollability) were risk factors for AC. Demonstrativeness often involves negative, destructive, antisocial tendencies, especially in the case of adolescents and young people, and requires increased prevention and correction efforts so that it does not become a core aspect of personality. Students with maladaptive personality structures may need to be oriented toward creative, dynamic means of socializing and spending time, or manifesting energy (e.g., team sports, bike riding, literary or art contests, dance, theater clubs, workshops on healthy lifestyles). Leisure education in a prosocial manner should start in childhood, both in the family and at school.

AC tends to be quite high in young adults in Romania, especially among men, peer groups, and in those who have problems socializing. Public health education programs should be targeted especially for these categories.

## Abbreviations

AC: Alcohol consumption
FACES IV: Family Adaptability and Cohesion Scale IV
NAA: National Anti-Doping Agency
SD: Standard deviation
WHO: World Health Organization
